# Involvement of DNA mismatch repair in the maintenance of heterochromatic DNA stability in *Saccharomyces cerevisiae*

**DOI:** 10.1371/journal.pgen.1007074

**Published:** 2017-10-25

**Authors:** Basanta K. Dahal, Lyudmila Y. Kadyrova, Kristin R. Delfino, Igor B. Rogozin, Vaibhavi Gujar, Kirill S. Lobachev, Farid A. Kadyrov

**Affiliations:** 1 Department of Biochemistry and Molecular Biology, Southern Illinois University School of Medicine, Carbondale, IL, United States of America; 2 Center for Clinical Research, Southern Illinois University School of Medicine, Springfield, IL, United States of America; 3 National Center for Biotechnology Information, National Library of Medicine, National Institutes of Health, Bethesda, MD, United States of America; 4 School of Biological Sciences and Institute for Bioengineering and Bioscience, Georgia Institute of Technology, Atlanta, GA, United States of America; National Institute of Environmental Health Sciences, UNITED STATES

## Abstract

Heterochromatin contains a significant part of nuclear DNA. Little is known about the mechanisms that govern heterochromatic DNA stability. We show here that in the yeast *Saccharomyces cerevisiae* (i) DNA mismatch repair (MMR) is required for the maintenance of heterochromatic DNA stability, (ii) MutLα (Mlh1-Pms1 heterodimer), MutSα (Msh2-Msh6 heterodimer), MutSβ (Msh2-Msh3 heterodimer), and Exo1 are involved in MMR at heterochromatin, (iii) Exo1-independent MMR at heterochromatin frequently leads to the formation of Pol ζ-dependent mutations, (iv) MMR cooperates with the proofreading activity of Pol ε and the histone acetyltransferase Rtt109 in the maintenance of heterochromatic DNA stability, (v) repair of base-base mismatches at heterochromatin is less efficient than repair of base-base mismatches at euchromatin, and (vi) the efficiency of repair of 1-nt insertion/deletion loops at heterochromatin is similar to the efficiency of repair of 1-nt insertion/deletion loops at euchromatin.

## Author summary

Eukaryotic mismatch repair is an important intracellular process that defends DNA against mutations. Inactivation of mismatch repair in human cells strongly increases the risk of cancer initiation and development. Although significant progress has been made in understanding mismatch repair at euchromatin, mismatch repair at heterochromatin is not well understood. Baker’s yeast is a key model organism to study mismatch repair. We determined that in baker’s yeast (1) mismatch repair protects heterochromatic DNA from mutations, (2) the MutLα, MutSα, MutSβ, and Exo1 proteins play important roles in mismatch repair at heterochromatin, (3) Exo1-independent mismatch repair at heterochromatin is an error-prone process; (4) mismatch repair cooperates with two other intracellular processes to protect the stability of heterochromatic DNA; and (5) the efficiency of repair of base-base mismatches at heterochromatin is lower than the efficiency of repair of base-base mismatches at euchromatin, but the efficiency of 1-nt insertion/deletion loop repair at heterochromatin is similar to the efficiency of 1-nt insertion/deletion loop repair at euchromatin.

## Introduction

Mutations fuel evolution and are also the basis of numerous diseases including cancer [1]. Base substitutions, 1-bp deletions, and 1-bp insertions are the most common mutations in the cell. Mutations are formed as a result of DNA damage and replication errors. Cells have evolved multiple mechanisms that suppress mutations [1, 2]. The high-fidelity DNA synthesis and DNA mismatch repair (MMR) play major roles in protecting the genome from mutations [3–8]. Replicative DNA polymerases achieve the high-fidelity DNA synthesis by selecting correct dNTPs and by proofreading DNA synthesis errors [4, 9]. The nucleotide selectivity step is responsible for keeping the error rate of replicative DNA synthesis at the level of 10^−4^–10^−6^, and proofreading further increases the fidelity of replicative DNA synthesis by 10–1,000 fold. Nuclear DNA is mainly synthesized by DNA polymerases (Pols) α, δ, and ε [10–12]. At the eukaryotic DNA replication fork, Pol ε performs the bulk of leading-strand synthesis, and Pol δ carries out the majority of lagging-strand synthesis [10–15]. Pol δ and Pol ε are the only two nuclear DNA polymerases that have proofreading activities [16, 17]. Pol δ can proofread both its own errors and those of Pol ε, but Pol ε can only proofread its own mistakes [18].

MMR removes a large fraction of DNA polymerase errors that escape proofreading [17, 19–21]. As a result, MMR decreases the level of spontaneous mutations in the genome by ~100 fold [22]. MMR efficiency is different at different genomic sites [23], and it is higher on the lagging than leading strand [24]. Strand breaks in the leading and lagging strands are likely to be the signals that direct eukaryotic MMR to remove mismatches on the daughter strands [25–28]. MutLα- and Exo1-dependent MMR is a major mechanism for correction of DNA polymerase errors in eukaryotic cells [29, 30]. This mechanism includes mismatch excision and DNA re-synthesis steps, and it involves a mismatch recognition factor (MutSα or MutSβ), the replicative clamp PCNA, the PCNA loader RFC, and Pol δ, in addition to MutLα endonuclease and the 5’→3’ exonuclease Exo1 [29–58]. Loss of Exo1 causes a modest defect in MMR, indicating that MMR is able to occur via Exo1-independent mechanism(s) [41, 45, 48, 57, 59–61]. Exo1-independent MMR is not as well understood as Exo1-dependent MMR [29, 30, 62]. A genetic study implicated proofreading activity of Pol δ in Exo1-independent MMR in budding yeast [45], and biochemical analyses of defined systems provided evidence that MutSα, MutLα endonuclease, PCNA, RFC, and Pol δ-driven strand displacement DNA synthesis are involved in human Exo1-independent MMR [57, 61].

Like proofreading and MMR, the histone acetyltransferase Rtt109 [63–65] is required for high-fidelity DNA replication [66]. Loss of Rtt109 increases the spontaneous mutation rate [66]. Rtt109 supports DNA replication fidelity by acetylating histone H3 on the K56 residue [66]. Histone H3 K56ac is an abundant histone modification associated with S phase and DNA replication in *S*. *cerevisiae* [67, 68]. The mechanism by which Rtt109-dependent H3 K56ac maintains the replication fidelity is not well understood, but a genetic analysis [66] indicated that it is likely to entail Rad51 and Rad52, key components of the homologous recombination machinery [69].

Nuclear DNA is packaged into euchromatin and heterochromatin soon after the passage of the DNA replication fork [70, 71]. Compared to euchromatin, heterochromatin is more condensed. Transcription in heterochromatin is silenced/suppressed whereas it is active in euchromatin. In *S*. *cerevisiae*, heterochromatin is present at *HMR*, *HML*, subtelomeric regions, and the rDNA locus [70, 72]. Sir2, Sir3, and Sir4 proteins are the structural components of heterochromatin at *HMR*, *HML*, and subtelomeric regions [70], but heterochromatin at rDNA does not include the latter two proteins. A Sir2-Sir3-Sir4-nucleosome complex is the basic unit of heterochromatin at the *HMR*, *HML*, and subtelomeric loci [70, 72, 73]. In this complex, Sir2-Sir3-Sir4 heterotrimer contacts the nucleosome via Sir3 and Sir4. In addition to being a structural component of yeast heterochromatin, Sir2 also has a NAD^+^-dependent histone deacetylase activity that is required for heterochromatin formation [74]. In the process of heterochromatin formation Sir2 deacetylates the N-terminal tails of nucleosomal histones H3 and H4, facilitating loading of Sir2-Sir3-Sir4 complexes onto the nucleosomes.

Previous research has been mainly focused on investigating eukaryotic MMR in the context of naked DNA and euchromatin. Up to date, only two studies have analyzed MMR at heterochromatin [75, 76]. One of the studies used bioinformatic approaches to investigate distribution of base-base substitutions at 1-Mb resolution in late heterochromatic and early euchromatic regions of cancer genomes [75]. It provided evidence that in cancer cells the MMR system removes base-base mismatches less efficiently at heterochromatin than at euchromatin. The other study revealed that Msh6-dependent correction of small insertion/deletion loops and base-base mismatches in *S*. *pombe* is less efficient at heterochromatin than at euchromatin [76]. In this study, we examined MMR at heterochromatin in *S*. *cerevisiae*. We determined that MMR at heterochromatin involves MutLα, MutSα, MutSβ, an Exo1 and that MMR occurring at heterochromatin in the absence of Exo1 is an error-prone process. In addition, we determined that MMR cooperates with the Pol ε proofreading activity and Rtt109 to maintain the stability of heterochromatic DNA. In agreement with a previous study [75], we established that the efficiency of repair of base-base mismatches at heterochromatin is lower than the efficiency of repair of base-base mismatches at euchromatin. However, we found that the efficiency of 1-nt insertion/deletion loop repair at heterochromatin is very similar to the efficiency of 1-nt insertion/deletion loop repair at euchromatin. This finding does not support the model that the efficiency of MMR at heterochromatin is reduced by lower accessibility of MMR proteins to heterochromatic DNA compared to euchromatic DNA [75, 76].

## Results

### Contribution of MMR to the maintenance of heterochromatic DNA stability in *S*. *cerevisiae*

**Fig 1:**
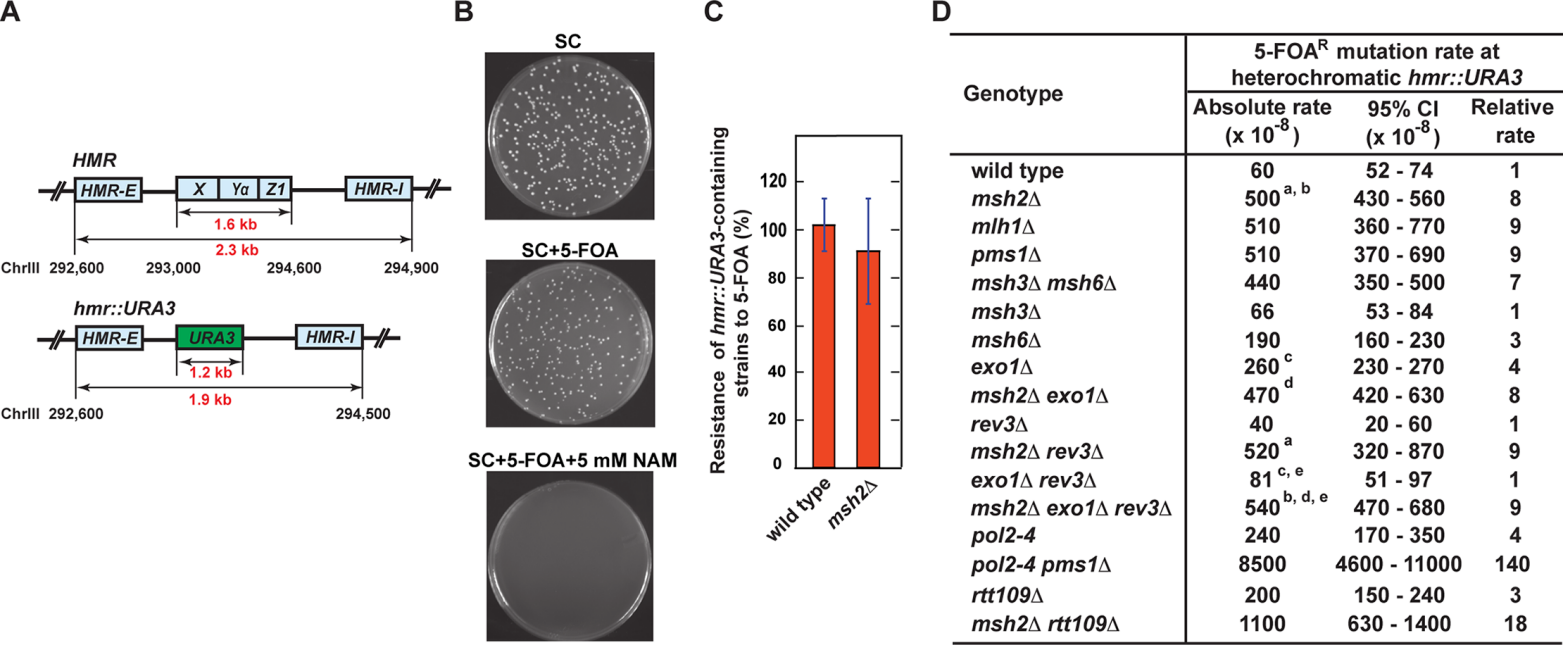
Importance of MMR for the stability of heterochromatic DNA at *hmr*::*URA3*. (**A**) Genetic maps of heterochromatic *HMR* and *hmr*::*URA3*. (**B**) *URA3* is heterochromatic at *hmr* in a wild-type strain. The same dilution of an overnight culture of the wild-type strain was plated onto a synthetic complete medium (SC), a SC medium supplemented with 1 g/L 5-FOA (SC + 5-FOA), and a SC medium supplemented with 1 g/L 5-FOA and 5 mM nicotinamide (SC + 5-FOA + 5 mM NAM). The plates were incubated at 30°C for 3 days. Representative images are shown. (**C**) Deletion of *MSH2* does not perturb heterochromatin at *hmr*::*URA3*. The experiments were carried out as described in **B**. The data are shown as averages ± 1 S.D. (n≥11). Additional analysis showed that heterochromatin at *hmr*::*URA3* is not affected by the *exo1Δ*, *msh2Δ exo1Δ*, *rev3Δ*, *msh2Δ rev3Δ*, *exo1Δ rev3Δ*, *mlh1Δ*, *pms1Δ*, *msh3Δ msh6Δ*, *msh3Δ*, *msh6Δ*, *pol2-4*, *pol2-4 pms1Δ*, *rtt109Δ*, and *msh2Δ rtt109Δ* mutations (**S1 Table**). (**D**) Spontaneous FOA^R^ mutation rates at heterochromatic *hmr*::*URA3* in the indicated isogenic strains. The wild-type strain was BKDY155. The mutation rates in this and other Figures and Tables are expressed as mutations per cell division and were measured as described under Materials and Methods. CI, confidence interval. The mutation rates that are marked with ^a^, ^b^, or ^d^ are not statistically different from each other (^a^p = 0.7, ^b^p = 0.19, and ^d^p = 0.21), and the mutation rates that are marked with ^c^ or ^e^ are statistically different from each other (^c^ p = 0.0001 and ^e^ p<0.0001).

**Fig 2:**
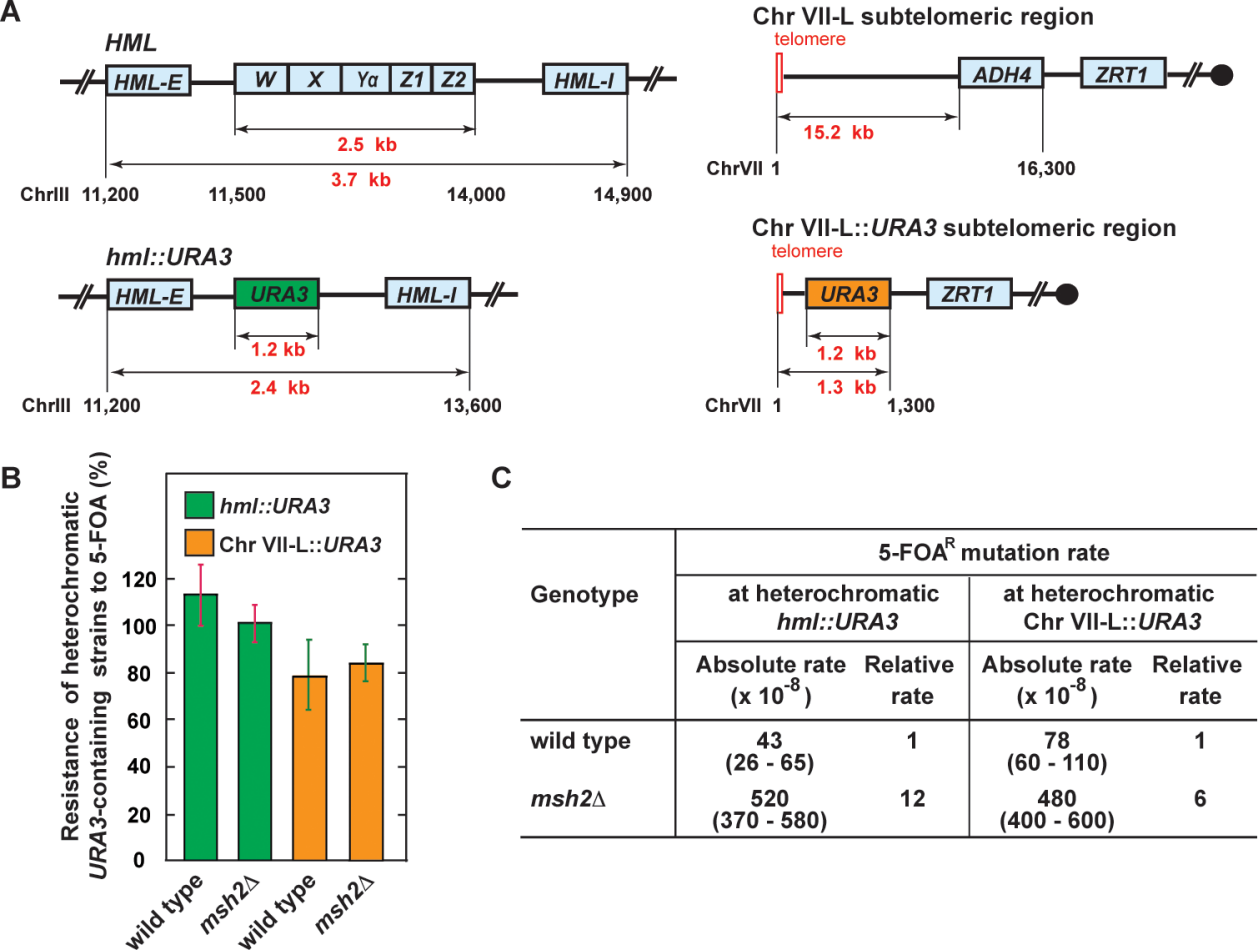
Contribution of MMR to the stability of heterochromatic DNA at *hml* and a Chr VII-L telomeric region. (**A**) Outline of heterochromatic *HML*, *hml*::*URA3*, Chr VII-L, and Chr VII-L::*URA3* regions. (**B**) Deletion of *MSH2* in the wild-type strains does not disrupt heterochromatin at the *hml*::*URA3* and Chr VII-L::*URA3* loci. The experiments were carried out as described in **Fig 1B**. The data are shown as averages ± 1 S.D. (n≥6). (**C**) Spontaneous mutation rates at the heterochromatic *hml*::*URA3* and Chr VII-L::*URA3* loci in the isogenic wild-type and *msh2Δ* strains. The wild-type strains were BKDY438 and BKDY541. The mutation rates were measured as described under Materials and Methods. 95% confidence intervals are in parentheses.

We started this work to study the impact of MMR on spontaneous mutation rates at heterochromatic loci in *S*. *cerevisiae*. In the majority of our experiments, we utilized a forward mutation assay that took advantage of the *URA3* gene. In this assay, yeast cells that acquire loss-of-function mutations in heterochromatic *URA3* are selected on a medium containing 5-FOA (5-fluoroorotic acid) and 5 mM nicotinamide (NAM). NAM was included into the selective medium because it switches the heterochromatic *URA3* to a euchromatic state, which leads to its expression [77, 78]. We first confirmed that when the *URA3* reporter was inserted at *hmr* (**Fig 1A**) in a wild-type strain, it was in a heterochromatic state (**Fig 1B**). This is in a full agreement with a previous work that showed that a similar reporter, *K*. *lactis URA3*, is heterochromatic at *hmr* [78]. We then established that *MSH2* was not required to maintain the heterochromatic status of *URA3* at *hmr* (**Fig 1C**). Next, we studied how *MSH2* deletion in the wild-type strain affected the 5-FOA^R^ mutation rate at heterochromatic *hmr*::*URA3*. As shown in **Fig 1D**, we found that deletion of *MSH2* in the wild-type strain increased the 5-FOA^R^ mutation rate at heterochromatic *hmr*::*URA3* by 8 fold. This finding showed that MMR was involved in the protection of heterochromatic *hmr*::*URA3* from mutations. We also investigated whether loss of *MSH2* affected mutation rates at two other heterochromatic loci: *hml*::*URA3* and Chr VII-L::*URA3* (**Fig 2**). (The latter locus is near the left telomere of Chr VII [79].) The data revealed that *MSH2* deletion in the wild-type strains increased the 5-FOA^R^ mutation rates at heterochromatic *hml*::*URA3* and *Chr VII-L*::*URA3* loci by 12 and 6 fold, respectively (**Fig 2**). Collectively, the results of these experiments demonstrated that MMR was essential for the maintenance of heterochromatic DNA stability in *S*. *cerevisiae*.

### Participation of MutLα, MutSα, and MutSβ in MMR at a heterochromatic locus

Yeast MutLα (Mlh1-Pms1 heterodimer), MutSα (Msh2-Msh6 heterodimer), and MutSβ (Msh2-Msh3 heterodimer) play important roles in MMR at euchromatic loci [31, 36, 80, 81]. We performed experiments to study whether these proteins contributed to MMR at heterochromatic *hmr*::*URA3*. We determined that the FOA^R^ mutation rate in the *mlh1Δ*, *pms1Δ*, or *msh3Δ msh6Δ* strain was similar to that in the *msh2Δ* strain (**Fig 1D**). Additionally, we determined that the FOA^R^ mutation rate for the *msh6Δ* strain was 3 times higher than that for the wild-type strain and that the *msh3Δ* strain displayed the same FOA^R^ mutation rate as the wild-type strain (**Fig 1D**). Collectively, these experiments revealed that (i) MutLα, MutSα, and MutSβ were involved in MMR at heterochromatin and (ii) MutSα played a more important role in MMR at heterochromatic *hmr*::*URA3* than MutSβ.

### Involvement of Exo1 in MMR at a heterochromatic locus

**Fig 3:**
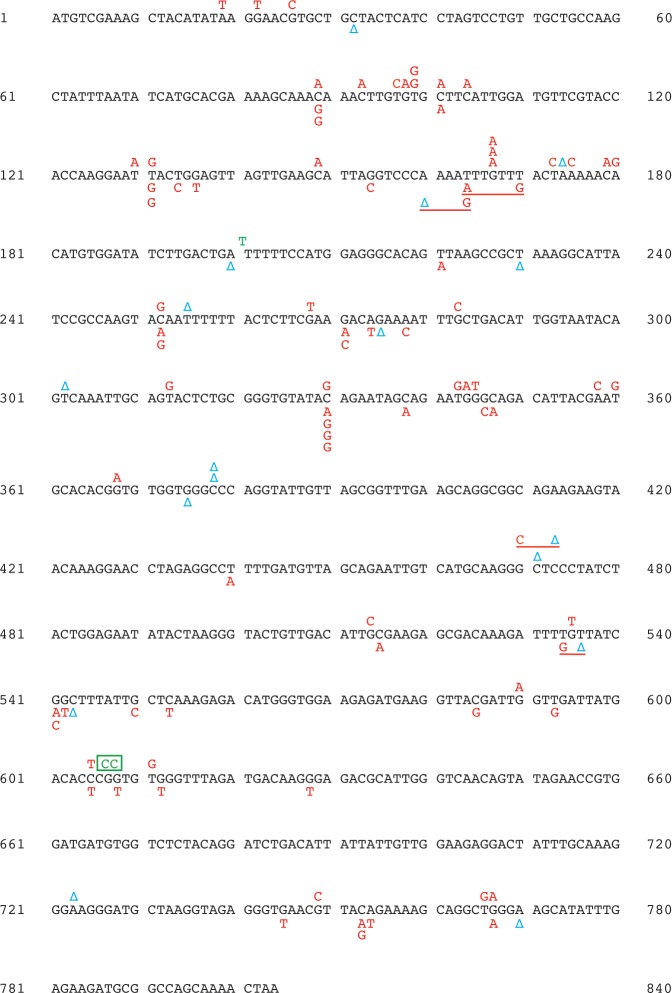
Spectra of *ura3* mutations at heterochromatic *hmr* in the wild type and *exo1Δ* strains. The entire sequence of the *URA3* open reading frame is shown. Characters above and below the *URA3* sequence represent *ura3* mutations at heterochromatic *hmr* in the wild type and *exo1Δ* strains, respectively. Base substitutions, 1-bp deletions, and 1-bp insertions are depicted as red capital letters, blue delta symbols, and green capital letters, respectively. A 2-bp deletion is boxed, and underlined symbols represent complex mutations. The mutation spectra were determined as described under Materials and Methods.

**Fig 4:**
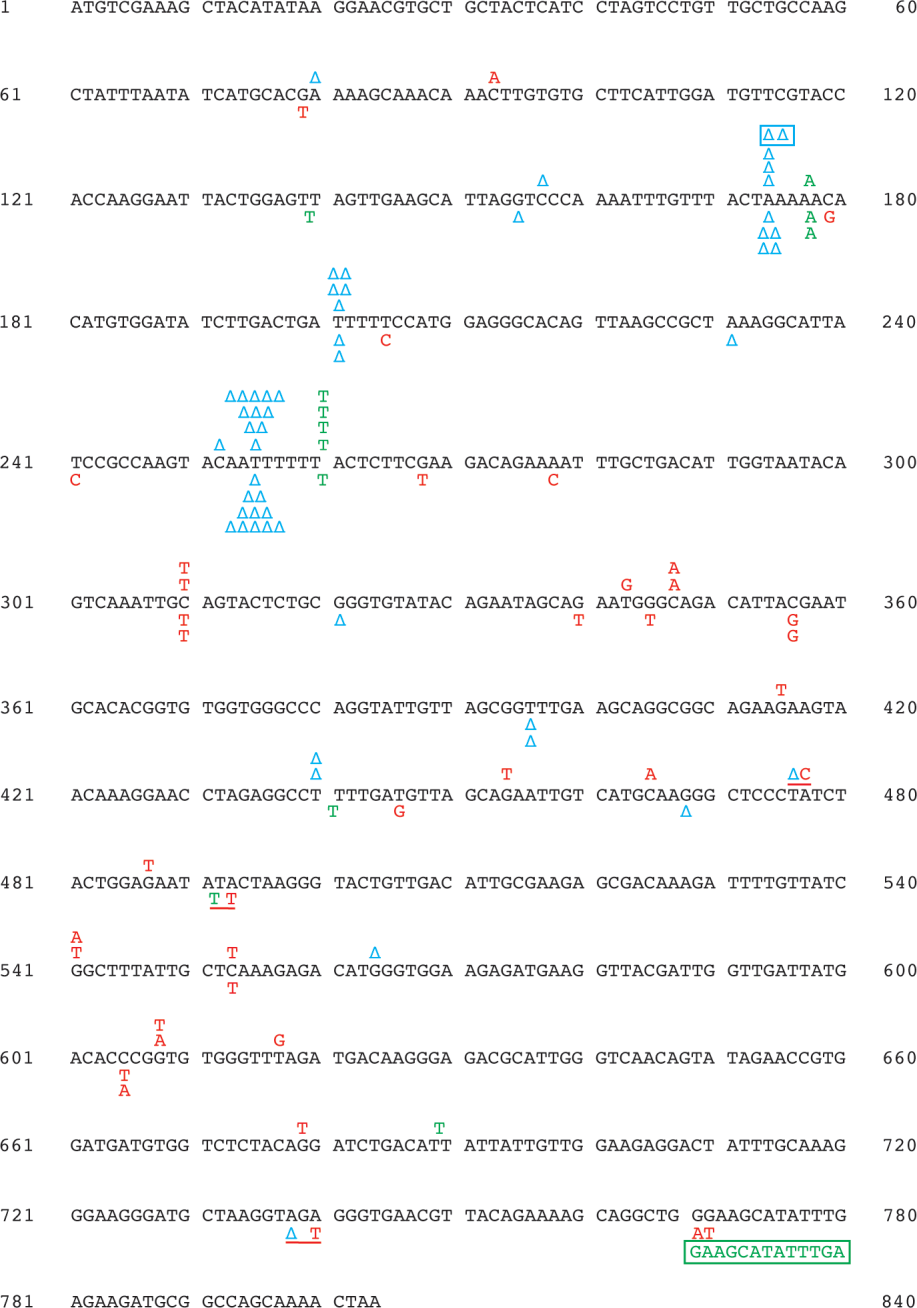
*ura3* mutation spectra at heterochromatic *hmr* in the *msh2*Δ and *msh2*Δ *exo1*Δ strains. Symbols above and below the *URA3* open reading frame denote *ura3* mutations at heterochromatic *hmr* in the *msh2*Δ and *msh2Δ exo1*Δ strains, respectively. Red capital letters, blue delta symbols, and green capital letters represent base substitutions, 1-bp deletions, and 1-bp insertions, respectively. A 2-bp deletion and a 13-bp duplication are boxed, and complex mutations are underlined.

Previous genetic studies implicated Exo1 in MMR at euchromatic regions [41, 48, 59, 60]. We studied whether Exo1 had a role in MMR at a heterochromatic locus. We established that deletion of *EXO1* in a wild-type strain increased the 5-FOA^R^ mutation rate at heterochromatic *hmr*::*URA3* by 4-fold and that *msh2Δ* was epistatic to *exo1Δ* for 5-FOA^R^ mutations at heterochromatic *hmr*::*URA3* (**Fig 1D**). These data suggested that loss of *EXO1* caused a strong defect in MMR at heterochromatin. We then determined and analyzed the *ura3* mutation spectra at heterochromatic *hmr* in the wild-type, *exo1Δ*, *msh2Δ*, and *msh2Δ exo1Δ* strains (**Table 1**, **Figs 3 and 4**). It can be seen that the most common mutations in the *ura3* mutation spectra of the wild-type and *exo1Δ* strains were base substitutions, whereas the most common mutations in the *ura3* mutation spectra of the *msh2Δ* and *msh2Δ exo1Δ* strains were 1-bp deletions. Further analysis of the data revealed that ~95% of 1-bp deletions in the *msh2Δ* and *msh2Δ exo1Δ* spectra were within N≥3 mononucleotide runs (**Fig 4**), but only ~60% and ~15% of 1-bp deletions in the wild-type and *exo1Δ* spectra, respectively, were within such runs (**Fig 3**). To determine whether the *ura3* mutation spectra of the *msh2Δ*, *msh2Δ exo1Δ*, *exo1Δ*, and wild-type strains were statistically different from each other or not, we performed the pairwise comparisons using χ2 test of independence and adjusted the p values with the Bonferroni correction. The data showed that there was no statistical difference between the *ura3* mutation spectra of the *msh2Δ* and *msh2Δ exo1Δ* strains, whereas those two spectra were statistically different from the *ura3* mutation spectra of the wild-type and *exo1Δ* strains (**Table 2**). In addition, we conducted the pairwise comparisons of the *ura3* mutation spectra of the *msh2Δ*, *msh2Δ exo1Δ*, and wild-type strains utilizing a Monte Carlo modification of the Pearson χ2 test of spectra homogeneity [82]. For this statistical analysis the spectra were arranged in a way (**S2 Table**) that was different from the one shown in **Table 1**. The results of this statistical analysis revealed that the *ura3* mutation spectra of the *msh2Δ* and *msh2Δ exo1Δ* strains were not statistically different from each other (χ^2^ = 7.9, P = 0.8945), but were statistically different from the *ura3* mutation spectrum of the wild-type strain (χ^2^ = 38.1 and 39.7, respectively, P < 10^−5^, the critical 5% value = 20.9). Our findings that the *ura3* mutation spectra of the *msh2Δ* and *msh2Δ exo1Δ* strains were not statistically different from each other and that *msh2Δ* was epistatic to *exo1Δ* with respect to FOA^R^ mutations at heterochromatic *hmr*::*URA3* (**Fig 1D**) demonstrated that Exo1 was involved in MMR at heterochromatin. Moreover, our finding that the *ura3* mutation spectrum of an *exo1Δ* strain was statistically different from the *ura3* mutation spectra of the *msh2Δ* and *msh2Δ exo1Δ* strains showed that Exo1-independent MMR at heterochromatin produced mutational intermediates.

**Table 1:** Spectra of *ura3* mutations at heterochromatic *hmr* in the wild-type, *msh2Δ, msh2Δ exo1Δ*, and *exo1Δ* strains.

Mutation type	Genotype			
	wild type	*msh2Δ*	*msh2Δ exo1Δ*	*exo1Δ*
1-bp deletions	7	25	24	7
Base substitutions	40	17	18	40
1-bp insertions	1	6	5	0
Complex mutations	1	1	2	3
Other mutations*	1	1	1	0
Total	50	50	50	50

The mutation spectra were obtained as described in Materials and Methods.

*, other mutations in the FOA^R^ mutation spectra of wild-type, *msh2Δ*, and *msh2Δ exo1Δ* strains were a 2-bp insertion, a 2-bp deletion, and a 13-bp duplication, respectively.

**Table 2:** Summary of χ2 test of independence of the *ura3* mutation spectra at heterochromatic *hmr*.

Comparison	p-value	p-values adjusted for 7 comparisons with the Bonferroni correction
Overall	< .0001	<0.007
wild type vs. *msh2Δ*	0.0001	0.0007
wild type vs. *msh2Δ exo1Δ*	0.0004	0.0028
wild type vs. *exo1Δ*	0.5578	~1
*msh2Δ* vs. *msh2Δ exo1Δ*	0.9761	~1
*msh2Δ* vs. *exo1Δ*	< .0001	<0.0007
*msh2Δ exo1Δ* vs. *exo1Δ*	< .0001	<0.0007

The mutation spectra used for the pairwise comparisons are shown in **Table 1**.

### Formation of *REV3*-dependent mutational intermediates during Exo1-independent MMR at a heterochromatic locus

**Fig 5:**
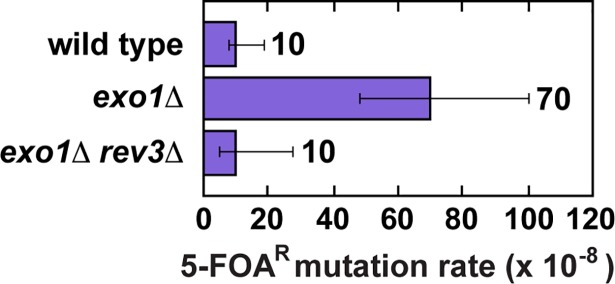
Effect of *REV3* deletion on the mutation rate at euchromatic ChrV::*URA3* in an *exo1*Δ strain. The mutation rates are presented as medians with 95% confidence intervals and were measured as described under Materials and Methods except that the selective medium for the mutation rate measurements was a SC medium containing 1 g/L 5-FOA. The *exo1*Δ and *exo1*Δ *rev3*Δ strains are isogenic derivatives of a wild-type strain (FKY1292).

The *REV3* gene encodes the catalytic subunit of the error-prone Pol ζ [83–85]. Prior work showed that deletion of *REV3* in an *exo1Δ* strain suppresses the mutation rate at euchromatic *CAN1* [81]. In agreement with this, we found that introduction of *rev3Δ* into an *exo1Δ* strain suppressed the mutation rate at euchromatic Chr V::*URA3* (**Fig 5**). To understand the origin of mutational intermediates, which arose at heterochromatic *hmr*::*URA3* as a result of Exo1-independent MMR, we carried out experiments to determine whether deletion of the *REV3* gene in the *exo1Δ*, *msh2Δ*, and *msh2Δ exo1Δ* strains affected the FOA^R^ mutation rates. These experiments demonstrated that deletion of *REV3* in the *exo1Δ* strain decreased the FOA^R^ mutation rate to the level observed in the wild-type strain, but deletion of *REV3* in the *msh2Δ* and *msh2Δ exo1Δ* strains did not change the FOA^R^ mutation rates (**Fig 1D**). Thus, FOA^R^ mutations produced at heterochromatic *hmr*::*URA3* in the *exo1Δ* strain were *REV3*-dependent, whereas FOA^R^ mutations produced at heterochromatic *hmr*::*URA3* in the *msh2Δ* and *msh2Δ exo1Δ* strains were *REV3*-independent. Based on these results, we concluded that Exo1-independent MMR at heterochromatin often produced Rev3-dependent mutational intermediates. Our analysis of the mutation rates in the *msh6Δ rev3Δ*, *msh6Δ exo1Δ rev3Δ*, *msh3Δ rev3Δ*, and *msh3Δ exo1Δ rev3Δ* strains was consistent with this conclusion (**S3 Table).**

### Cooperation of the MMR system with Pol ε proofreading and the histone acetyltransferase Rtt109 in the maintenance of heterochromatic DNA stability

The MMR system removes DNA polymerase errors at euchromatic loci [17, 19–21, 45, 86]. To examine whether the MMR system removes Pol ε errors at a heterochromatic locus, we constructed an *hmr*::*URA3 pol2-4 pms1Δ* strain. (*pol2-4* encodes the catalytic subunit of Pol ε, which lacks the proofreading activity [16].) In agreement with the previous work [17], we noticed that the *pol2-4 pms1Δ* mutant grew poorly and single colonies of this mutant were of different sizes (small, medium and large). Our analysis showed that the relative mutation rate in the *pol2-4 pms1Δ* double mutant was 11 times higher than the sum of the relative mutation rates in the single mutants (i.e. there was a strong synergistic relationship between *pol2-4* and *pms1Δ* for FOA^R^ mutations at heterochromatic *hmr*::*URA3*) (**Fig 1D**). The presence of the strong synergistic relationship demonstrated that at heterochromatic *hmr*::*URA3* Pol ε errors that were not removed by its proofreading activity were corrected by the MMR system.

The MMR system and histone acetyltransferase Rtt109 act in overlapping pathways to preserve the replication fidelity at euchromatic sites [66]. We explored whether a similar cooperation between the MMR system and Rtt109 took place at heterochromatic *hmr*::*URA3*. The experiments revealed that there was a weak synergistic relationship between *msh2Δ* and *rtt109Δ* for *ura3* mutations at heterochromatic *hmr*::*URA3* (**Fig 1D**). To better understand the nature of the cooperation, we determined and analyzed the spectra of *ura3* mutations at heterochromatic *hmr* in the *rtt109Δ* and *msh2Δ rtt109Δ* strains (**S1 Fig** and **Table 3**). The data indicated that the MMR system and Rtt109 acted in overlapping pathways that increased the replication fidelity by suppressing base substitutions and 1-bp deletions.

**Table 3:** Rates of different types of *ura3* mutations at the heterochromatic *hmr* locus in the indicated *S*. *cerevisiae* strains.

Genotype	FOA^R^ mutation rate (x 10^−8^) at heterochromatic *hmr*::*URA3*					
	Base substitutions	1-bp deletions	1-bp insertions	Complex mutations	Other mutations	Total
Wild type	48	8.4	1.2	1.2	1.2	60
(n = 50)	(1)	(1)	(1)	(1)	(1)	(1)
*msh2Δ*	170	250	60	10	10	500
(n = 50)	(3.5)	(30)	(50)	(8.3)	(8.3)	(8.3)
*rtt109Δ*	156	13	5	18	9	200
(n = 45)	(3.3)	(1.6)	(4.2)	(15)	(7.5)	(3.3)
*rtt109Δ msh2Δ*	420	590	46	<23	46	1,100
(n = 47)	(8.8)	(70)	(38)	(<19)	(38)	(18)

The mutation spectra were obtained as described in Materials and Methods. The relative mutation rates are in parentheses.

### Reduced efficiency of *MSH2*-dependent repair of base-base mismatches at a heterochromatic locus

**Fig 6:**
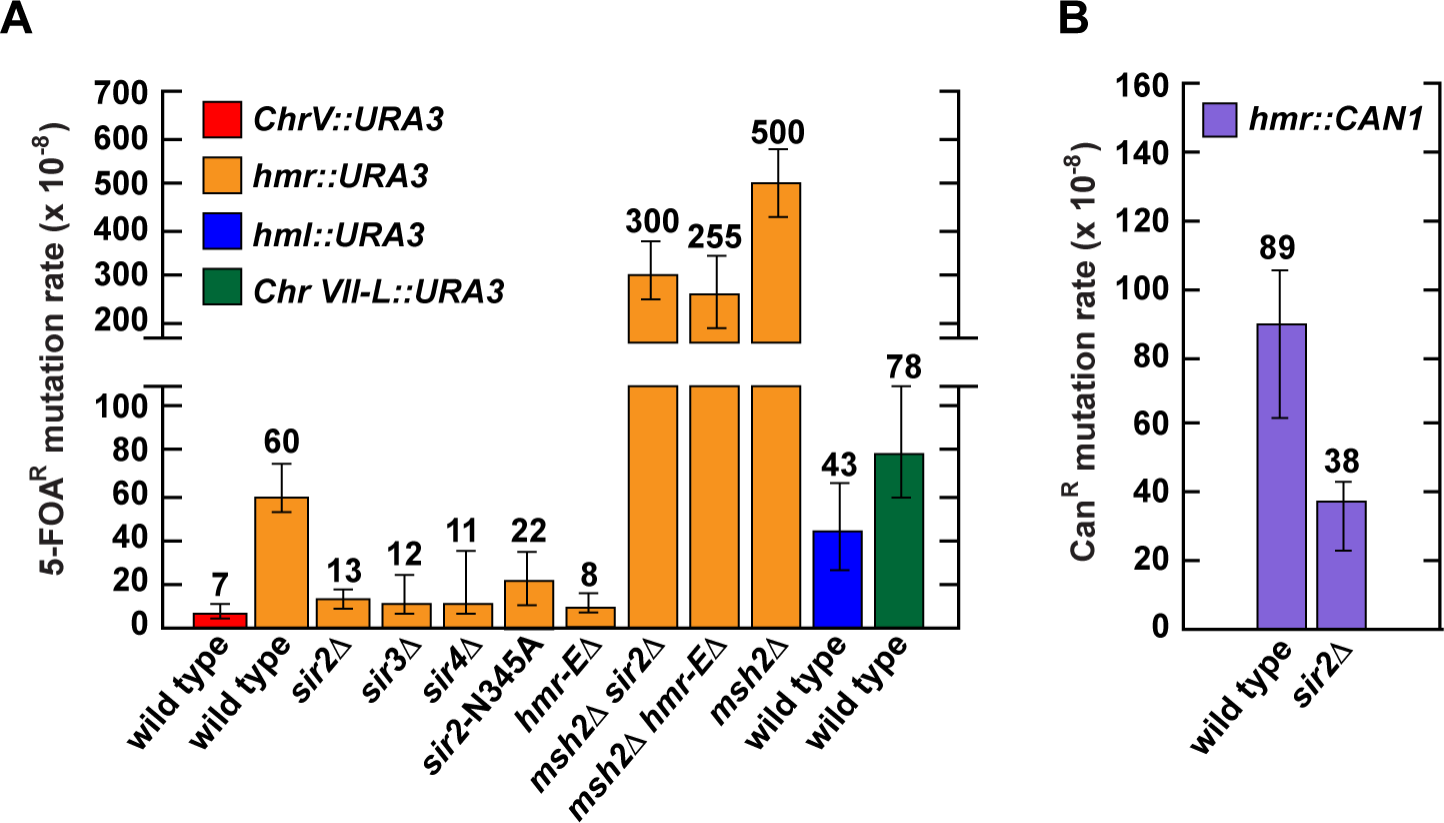
Impact of heterochromatin environment on the mutation rates. The FOA^R^ (**A**) and Can^R^ (**B**) mutation rates are shown as medians with 95% confidence intervals. The mutation rates were determined as described under Materials and Methods.

During the course of this work, we noticed that the mutation rates at heterochromatic *hmr*::*URA3*, *hml*::*URA3*, and Chr VII-L::*URA3* in our wild-type strains (**Figs 1 and 2**) were 2–4 times higher than the mutation rates at euchromatic *CAN1* in other wild-type strains [45, 66, 80]. To ensure that the observed difference in the mutation rates was not a result of genetic background variations and/or the use of the different mutation reporters, we inserted *URA3* at a Chr V euchromatic locus (where it is normally located) in a wild-type strain, which was isogenic to the strains carrying the heterochromatic reporters (**Figs 1 and 2**), and measured an FOA^R^ mutation rate in this strain. As shown in **Fig 6A**, the mutation rate at euchromatic Chr V::*URA3* of this wild-type strain was ~ 6–11 times lower than a mutation rate at a heterochromatic locus of a similar wild-type strain. This observation was consistent with an idea that in wild-type strains, heterochromatic DNA was less stable than euchromatic DNA. To test this idea we disrupted heterochromatin by introduction of *sir2Δ*, *sir3Δ*, *sir4Δ*, *sir2-N345A*, or *hmr-EΔ* [87] mutation into a wild-type strain and measured the mutation rates in the constructed strains (**Fig 6A**). (The N345A mutation inactivates the Sir2 histone deacetylase activity, which is required for heterochromatin formation [74].) Analysis of the data showed that the mutation rate at *hmr*::*URA3* in *sir2Δ*, *sir3Δ*, *sir4Δ*, *sir2-N345A*, or *hmr-EΔ* strain was 3–7 times lower than the mutation rate at heterochromatic *hmr*::*URA3* in the wild-type strain (**Fig 6A**). These findings provided a strong support for the idea that in wild-type strains, heterochromatic DNA was less stable than euchromatic DNA. Additional support for this idea was obtained in experiments in which we established that the mutation rate at euchromatic *hmr*::*CAN1* in a *sir2Δ* strain was half that at heterochromatic *hmr*::*CAN1* in a wild-type strain (**Fig 6B**).

A previous elegant study demonstrated that the efficiency of *MSH2*-dependent repair of small insertion/deletion loops varies across the yeast genome [23]. MMR efficiency variations are likely to have important evolutionary consequences. In light of this information, we calculated MMR efficiencies at the heterochromatic and euchromatic loci. The calculated efficiencies of MMR at heterochromatic *hmr*::*URA3*, *hml*::*URA3*, and Chr VII-L::*URA3* were 88%, 92%, and 84%, respectively, and the calculated efficiency of MMR at euchromatic Chr V::*URA3* was 97%. Thus, these data suggested that MMR was less efficient at heterochromatin than euchromatin.

We next determined that (i) the efficiencies of repair of base-base mismatches and 1 nt-insertion/deletion loops at heterochromatic *hmr*::*URA3* were 72% and 97–98%, respectively, and (2) the efficiencies of repair of base-base mismatches and 1 nt-insertion/deletion loops at euchromatic Chr V::*URA3* were 96% and ~98–99%, respectively (**Table 4**). Based on these data, we concluded that the efficiency of repair of base-base mismatches at heterochromatic *hmr*::*URA3* was significantly reduced compared to the efficiency of repair of base-base mismatches at euchromatic Chr V::*URA3*.

**Table 4:** Efficiencies of repair of different types of mismatches at heterochromatic *hmr*::*URA3*, a euchromatic Chr V::*URA3*, and euchromatic *hmr::URA3* locus.

Location	Repair efficiency (%)			
	Base-base mismatches	1-nt deletion loops	1-nt insertion loops	Total
Heterochromatic *hmr*::*URA3* in a wild-type strain	72	97	98	88
Euchromatic Chr V::*URA3* in a wild-type strain	96	99	98	97
Euchromatic *hmr*::*URA3* in a *sir2Δ* strain	90	99.8	98.8	96

The repair efficiencies in the wild-type strains were calculated using the following formula: repair efficiency (%) = 100 –(100 x μ_wt_/μ_*msh2*Δ_), where μ_wt_ and μ_*msh2*Δ_ are rates of relevant types of mutations in the wild-type and *msh2*Δ strains, respectively. The repair efficiency in the *sir2*Δ strain was calculated using a similar formula: repair efficiency (%) = 100 –(100 x μ_*sir2*Δ_/μ_*msh2*Δ_ _*sir2*Δ_), where μ_*sir2*Δ_ and μ_*msh2*Δ_ _*sir2*Δ_ are rates of relevant types of mutations in the *sir2*Δ and *msh2*Δ *sir2*Δ strains, respectively. Rates of the different mutation types in all these strains are shown in **S4 Table**.

We thought that the reduced efficiency of repair of base-base mismatches at heterochromatic *hmr*::*URA3* might be a consequence of the heterochromatic environment. We reasoned that if this idea was correct, then disruption of heterochromatin at *hmr*::*URA3* by deletion of *SIR2* should increase the efficiency of repair of base-base mismatches at this locus. Our experiments showed that the efficiency of repair of base-base mismatches at euchromatic *hmr*::*URA3* in a *sir2Δ* strain was 90% (**Table 4**). Thus, disruption of heterochromatin at *hmr*::*URA3* by *sir2Δ* mutation increased the efficiency of repair of base-base mismatches at this locus from 72% to 90%. This observation suggested that the heterochromatic environment decreased the efficiency of repair of base-base mismatches.

## Discussion

### MMR is required for the stability of heterochromatic DNA in *S*. *cerevisiae*

Significant progress has been made in understanding of MMR at euchromatin since the demonstration of its importance for euchromatic DNA stability [31, 88, 89]. However, much less is known about MMR at heterochromatin [75, 76]. In this work, we have found that inactivation of MMR in *S*. *cerevisiae* significantly increases the spontaneous mutation rates at heterochromatic *hmr*::*URA3*, *hml*::*URA3*, and Chr VII-L::*URA3* loci (**Figs 1 and 2**). These findings have demonstrated that MMR is essential for the maintenance of heterochromatic DNA stability in *S*. *cerevisiae*. Furthermore, our analysis of the genetic interactions has provided strong evidence that in budding yeast MMR cooperates with Pol ε proofreading and Rtt109 to protect heterochromatic DNA from spontaneous mutations (**Fig 1D**).

Previous studies revealed that at euchromatic *CAN1* the mutation rates in *msh2Δ* strains are 27–40 times as high as those in wild-type strains [45, 66, 80]. Consistent with those studies, we have established that at a different euchromatic locus, Chr V::*URA3*, the mutation rate in an *msh2Δ* strain is 37 times higher than that in a wild-type strain (**S4 Table**). However, at each of the three heterochromatic loci, the mutation rate in the *msh2Δ* strain is only 6–12 times that of the wild-type strain (**Figs 1 and 2**). Thus, the budding yeast data substantiate the view that MMR is more important for euchromatic DNA stability than for heterochromatic DNA stability [75, 76]. The experiments, in which we have determined that at euchromatic *hmr*::*URA3* the mutation rate in an *msh2Δ sir2Δ* strain is 23 times that in an *sir2Δ* strain, have provided a direct support for such a view (**Fig 6A**).

### MutLα, MutSα, MutSβ, and Exo1 are involved in MMR at heterochromatin

Our data have implicated MutLα, MutSα, and MutSβ in MMR at heterochromatin (**Fig 1D**). MutLα, MutSα, and MutSβ are also involved in MMR at euchromatin [31, 41, 44, 48, 80, 90]. Thus, it appears that the roles of MutLα, MutSα, and MutSβ at heterochromatin are not very different from those at euchromatin [29, 30, 57].

Biochemical studies with cell-free extracts and reconstituted systems demonstrated the importance of the 5’-3’ exonuclease Exo1 for the mismatch excision step in the process that repairs base-base mismatches and 1-nt insertion/deletion loops [46–49, 91]. Such a role for Exo1 in MMR is in full agreement with genetic analyses of this process at euchromatic sites in yeast and mice [35, 41, 45, 48]. However, the importance of Exo1 for MMR at euchromatin was brought into question by the finding that the mutator phenotype of a yeast *exo1Δ* strain was not consistent with an MMR defect [81]. We have conducted experiments to investigate whether Exo1 plays a role in MMR at heterochromatin. In these experiments we have found that (i) at heterochromatic *hmr*::*URA3* the mutation rate in an *exo1Δ* strain is 4 times that in the wild-type strain and half that in the *msh2Δ* strain; (ii) deletion of *MSH2* is epistatic to deletion of *EXO1* for spontaneous FOA^R^ mutations at heterochromatic *hmr*::*URA3* (**Fig 1D**); and (iii) the *ura3* mutation spectrum at heterochromatic *hmr* in an *msh2Δ exo1Δ* strain is not statistically different from the *ura3* mutation spectrum at the same locus in an *msh2Δ* strain (**Table 2** and text in Results section). Collectively, these findings have shown that Exo1 plays a major role in MMR at heterochromatin.

### Exo1-independent MMR at heterochromatin is an error-prone process

**Fig 7:**
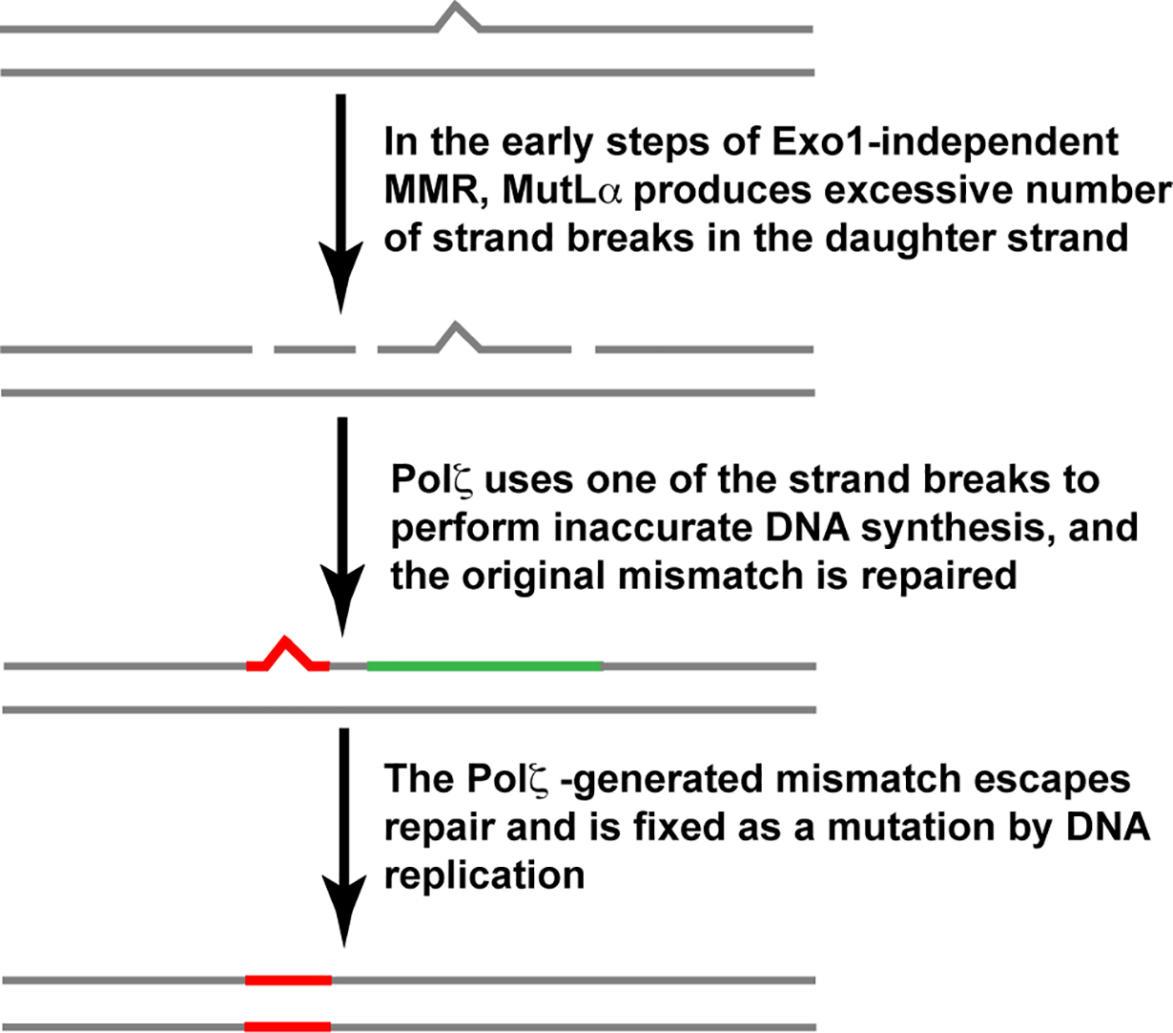
A model for eukaryotic Exo1-independent MMR that leads to the formation of *REV3*-dependent mutations. In this model, DNA polymerase errors are depicted as bumps in the top strand.

We have determined that (i) the mutation rate at heterochromatic *hmr*::*URA3* in an *exo1Δ* strain is *REV3*-dependent whereas the mutation rate at heterochromatic *hmr*::*URA3* in an *msh2Δ* strain is *REV3*-independent (**Fig 1D**) and (ii) the mutation spectrum at heterochromatic *hmr*::*URA3* in an *exo1Δ* strain is statistically different from the mutation spectra at heterochromatic *hmr*::*URA3* in the *msh2Δ* and *msh2Δ exo1Δ* strains (**Table 2**). Furthermore, it can be seen that the majority of 1-bp deletions in the spectra of the *msh2Δ* and *msh2Δ exo1Δ* strains are within the N≥5 mononucleotide runs (**Fig 4**), whereas not a single 1-bp deletion in the mutation spectrum of the *exo1Δ* strain is in any of these runs. These findings have indicated that Exo1-independent MMR at heterochromatin is an error-prone process that leads to the formation of Pol ζ-dependent mutations (**Fig 7**). Because Exo1 plays more important role in MMR on the lagging strand [92, 93], this error-prone process is likely to preferentially occur on the lagging than leading strand.

We would like to note that it has been suggested that the mutator phenotype of *exo1Δ* strains reveals the participation of Exo1 in both MMR and an MMR-unrelated mutation avoidance pathway [81]. However, if Exo1 participated in an MMR-unrelated mutation avoidance pathway functioning across the genome, then a mutation spectrum at a heterochromatic site in an *msh2Δ exo1Δ* strain should have been different from a mutation spectrum at the same site in an *msh2Δ* strain. In contrast, we determined that the mutation spectrum at heterochromatic *hmr*::*URA3* in an *msh2Δ exo1Δ* strain is not statistically different from the mutation spectrum at the same locus in an *msh2Δ* strain (**Table 2**).

We propose that error-prone Exo1-independent MMR at heterochromatin consists of three principal steps (**Fig 7**). In the initial step, Exo1-independent MMR at heterochromatin leads to the formation of an excessive number of MutLα endonuclease-dependent strand breaks in the discontinuous daughter strand. This happens because mismatch removal step in Exo1-independent MMR is slowed down by the absence of Exo1, which permits MutLα to produce additional strand breaks in the discontinuous daughter strand. Next, one of these strand breaks is used by Pol ζ (REV3-REV7-Pol31-Pol32 complex [94, 95]) to introduce a mismatch, and the original mismatch is corrected. Finally, the Pol ζ-produced mismatch escapes correction, perhaps due to the presence of an MMR impediment, and then is fixed as a mutation in the next round of DNA replication. We believe that this model also provides a satisfactory explanation for the formation of *REV3*-dependent mutations at euchromatin in *exo1Δ* strains [81] (**Fig 5**). Although we do not know what could block removal of the Pol ζ-produced mismatch by the Exo1-lacking MMR system, past work revealed that the nucleosome is able to function as an MMR impediment [96, 97]. Our experiments have shown that the mutator phenotypes of the *msh3Δ exo1Δ* and *msh6Δ exo1Δ* strains are *REV3*-dependent (**S3 Table**) and the mutator phenotype of the *msh2Δ exo1Δ* strain is *REV3*-independent (**Fig 1D**). Taken together, these data have demonstrated that Exo1-independent MutSα-dependent MMR at heterochromatin in *msh3Δ exo1Δ* cells and Exo1-independent MutSβ-dependent MMR at heterochromatin in *msh6Δ exo1Δ* cells often causes the formation of *REV3*-dependent mutations.

Wei et al. (2003) found that loss of Exo1 predisposes mice to the development of lymphomas [48]. That finding allowed the authors to propose that *EXO1* mutations may predispose humans to cancer [48]. However, strong evidence to support this proposal is still missing [98, 99]. In yeast, *EXO1* deficiency increases the mutation rates in the forward mutation assays to the levels that are 70–140% of those caused by *MSH6* deficiency [45] (**Fig 1D**). Based on these data, we envision that cancers triggered by *EXO1* mutations may be nearly as common as those initiated by *MSH6* mutations [100]. Previous work and our mutation spectrum data suggest that cancers triggered by *EXO1* deficiency will rarely display microsatellite instability [41, 45, 60] (**Figs 3 and 4**), which is a hallmark of MMR deficiency caused by *MSH2*, *MLH1*, or *PMS2* inactivation. Thus, to better understand the relationship between *EXO1* and cancer, it may be necessary to analyze microsatellite-stable, but not microsatellite-unstable, cancers that display increased mutation rates.

### Heterochromatic environment decreases the efficiency of *MSH2*-dependent repair of base-base mismatches

We have measured mutation rates at several genomic sites; one of the sites is euchromatic and the others are heterochromatic. We have found that the mutation rate at euchromatic Chr V::*URA3* in a wild-type strain is ~9, ~6, and ~11 times lower than the mutation rates at heterochromatic *hmr*::*URA3*, *hml*::*URA3*, and Chr VIIL::*URA3*, respectively, in similar wild-type strains (**Fig 6A**). Moreover, we have found that disruption of heterochromatin in a wild-type strain by *sir2Δ*, *sir3Δ*, *sir4Δ*, *sir2-N345A*, or *hmr-EΔ* [87] (**S1 Table**) decreases the mutation rate at *hmr* 2–7 fold (**Fig 6**). Together, these data have shown that in *S*. *cerevisiae* the heterochromatic DNA is less stable than the euchromatic DNA, which supports the idea that the chromatin environment is a key factor that affects the stability of DNA [23, 101].

Sun et al. (2016) have recently described that in an *S*. *pombe msh6Δ* strain mutation rate in heterochomatin is ~50% higher that in euchromatin [76]. Consistent with this, we have found that the mutation rate at heterochromatic *hmr*::*URA3* in the *msh2Δ* strain is 1.5–2 times higher than those at euchromatic *hmr*::*URA3* in the *msh2Δ hmr-EΔ* and *msh2Δ sir2Δ* strains (**Fig 6A**). Together, these findings suggest that the heterochromatic environment modestly increases the level of DNA replication errors at heterochromatic sites.

Our data (**Figs 1–3** and **Table 4**) corroborate the view that MMR efficiency varies from one locus to another and is an important factor that contributes to locus-specific mutation rates [23, 75]. Surprisingly, at heterochromatic *hmr*::*URA3* the efficiency of repair of base-base mismatches is only 72% but the efficiency of repair of 1-nt insertion/deletion loops is 97–98% (**Table 4**). Thus, the heterochromatic environment decreases the efficiency of repair of base-base mismatches but has a little of influence on the efficiency of repair of 1-nt deletion loops (**Table 4**). This finding argues against the model that the efficiency of MMR at heterochromatin is reduced by lower accessibility of MMR proteins to heterochromatic DNA compared to euchromatic DNA [75, 76]. We do not know how the heterochromatic environment reduces the efficiency of repair of base-base mismatches. It is likely that the overall efficiency of repair of base-base mismatches at heterochromatin is decreased because many base-base mismatches in newly replicated heterochromatic DNA are poor substrates for the MMR reaction. We envision that these base-base mismatches are poor substrates for the MMR reaction because they contain damaged bases, which arise as a result of low level of base excision repair at heterochromatin.

In summary, we have performed a detailed analysis of MMR at heterochromatin. Our research has demonstrated that MMR involves MutLα, MutSα, MutSβ, and Exo1 to maintain heterochromatic DNA stability. Surprisingly, it has also revealed that Exo1-independent MMR at heterochromatin is an error-prone process and that the repair of 1-nt insertion/deletion loops at heterochromatin is nearly as efficient as the repair of 1-nt insertion/deletion loops at euchromatin.

## Materials and methods

### Yeast strains and plasmids

The yeast *S*. *cerevisiae* strains are derivatives of BY4742 (*MAT*α *his3*Δ*1 leu2*Δ*0 lys2*Δ*0 ura3*Δ*0)*. The wild-type strains are BKDY155 (*MAT*α *his3*Δ*1 leu2*Δ*0 lys2*Δ*0 ura3*Δ*0 hmr*::*URA3 hml*::*HphMX*), BKDY157 (*MAT*α *his3*Δ*1 leu2*Δ*0 lys2*Δ*0* Chr V::*URA3 hml*::*HphMX*), BKDY438 (*MAT*α *his3*Δ*1 leu2*Δ*0 lys2*Δ*0 ura3*Δ*0 hml*::*URA3*), BKDY541 (*MAT*α *his3*Δ*1 leu2*Δ*0 lys2*Δ*0 ura3*Δ*0* Chr VII-L::*URA3*), BKDY834 (*MAT*α *his3*Δ*1 leu2*Δ*0 lys2*Δ*0 ura3*Δ*0 can1*::*LEU2 hmr*::*CAN1*), and FKY1292 (*MAT*α *his3*Δ*1 leu2*Δ*0 lys2*Δ*0* Chr V::*URA3*). In BKDY157 and FKY1292, a DNA sequence between nucleotides 115,949 and 117,045 of Chr V is replaced with *URA3*. Each of the mutant strains is isogenic to one of the wild-type strains. In the *hmr-E*Δ strain, the 56-bp *HMR-E* region (Chr III 292,674–292,729) [87] was replaced with a *LEU2* cassette. To create the gene deletions, PCR-amplified disruption cassettes were introduced into yeast cells by lithium acetate/PEG4000/DMSO transformation. The presence of each gene deletion was confirmed by locus and disruption cassette-specific PCRs. The *pol2-4* mutation was introduced into the chromosomal *POL2* gene using the integration-excision method, and the *sir2-N345A* mutation was inserted into the chromosomal *SIR2* gene utilizing a previously described technique [102].

### Measurements of mutation rates

The spontaneous mutation rates were measured using a fluctuation test. At least 9–18 cultures, which were started from single colonies of two-four independent isolates of the same genotype, were used to determine the spontaneous mutation rate. The cultures were grown to saturation in 3 ml YPDAU medium (1% yeast extract, 2% bacto-peptone, 2% dextrose, 60 mg/L adenine, 60 mg/L uracil) at 30°C. The saturated cultures were diluted in sterile water, and appropriate dilutions were plated on a synthetic complete (SC) medium to determine the total number of cells in the cultures and on a selective medium to determine the total number of the mutant cells in the cultures. Unless noted otherwise, the selective medium for FOA^R^ cells was a SC medium containing 1 g/L 5-FOA and 5 mM NAM (SC + 5-FOA + NAM), and the selective medium for Can^R^ cells was a SC medium that lacked arginine and contained 60 mg/L L-canavanine and 5 mM NAM. The plates were incubated for 3–4 days at 30°C, and the colonies were counted. 5–70% of the FOA^R^ colonies grew on a SC—Ura + 5 mM NAM medium. FOA^R^ cells, which formed these colonies, were excluded from calculations of the mutation rates. To identify FOA^R^ colonies that were Ura^+^, FOA^R^ colonies formed on the fluctuation test plates were replica-plated onto the SC—Ura + 5 mM NAM medium, and the plates were incubated for 1 day at 30°C.

The mutation rates were calculated from the total numbers of cells and mutants in the cultures using the Drake’s formula *μ* = *ƒ*/ln(*Nμ*) [103], where μ is mutation rate per replication, ƒ is the median mutant frequency, and *N* is population size.

### Statistical tests

Where indicated, the significance of the observed differences in the mutation rates was assessed with the Mann-Whitney U two-tailed test (GraphPad Prism 6 software), in which the null hypothesis is that there is no difference between the two data sets.

To examine the relationship between the nominal variables of spectra and mutation type (**Table 2**), categorical variables were summarized with frequencies and percentages, and a χ2 test of independence with a Bonferroni correction for multiple comparisons was utilized.

In a different method, a Monte Carlo modification of the Pearson χ2 test of spectra homogeneity [82] was used to compare mutation distributions (**S2 Table**). The calculations were done using the COLLAPSE program [104].

### Determination of *ura3* mutation spectra

In order to determine the *ura3* mutation spectrum, ~50–100 patches each started from a different single colony were grown on YPDAU plates (1% yeast extract, 2% bacto-peptone, 2% dextrose, 60 mg/L adenine, 60 mg/L uracil, 2% agar). The patches were next replica-plated on the SC + 5-FOA + 5 mM NAM, followed by incubation of the plates for 1 day at 30°C. The patches that were formed on the SC + 5-FOA + 5 mM NAM plates were replica-plated on fresh SC + 5-FOA + 5 mM NAM plates, and the plates were incubated for 2–3 days at 30°C. A single FOA^R^ colony was randomly selected from each patch, purified on a SC + 5-FOA + 5 mM NAM plate, and propagated on a YPDAU plate. The patches were then replica-plated on SC—Ura + 5 mM NAM plates. Patches that grew on the SC—Ura + 5 mM NAM plates were not analyzed further. Genomic DNAs of the remaining FOA^R^ patches were isolated with a MasterPure Yeast DNA purification kit (Epicentre). Each of these genomic DNAs was used as a template to PCR-amplify a 1.4-kb DNA fragment encompassing the entire length of *ura3* ORF with primers #1 (5’- GAGAATAAGCGCAGGTACTCCTG -3’) and #2 (5’- CGCCATATACGAAAATGTTGGTG -3’). The amplified DNA fragments were purified with a PCR purification kit (Thermo Fisher) and sequenced to determine *ura3* mutations.

## Supporting information

S1 Table Resistance of the indicated *hmr*::*URA3* strains to 5-FOA. The data are shown as averages ± 1 S.D. (n≥ 4).
(DOC)

S2 Table *ura3* mutation spectra at heterochromatic *hmr* in the indicated yeast strains. The mutation spectra were obtained as described in Materials and Methods.
(DOC)

S3 Table Mutation rates at heterochromatic *hmr*::*URA3* in the indicated strains. The strains are BKDY155 (wild type) and its mutant derivatives. The difference between mutation rates marked ^a^ or ^b^ is not statistically significant (^a^p = 0.6 and ^b^p = 0.15 in two-tailed Mann-Whitney test). 95% confidence intervals are in parentheses.
(DOC)

S4 Table Rates of different types of *ura3* mutations at the heterochromatic *hmr*, euchromatic Chr V, and heterochromatic loci. The mutation spectra were obtained as described in Materials and Methods. Each of the four spectra is composed of 50 mutations. 95% confidence intervals are in parentheses.
(DOC)

S1 Fig Spectra of *ura3* mutation at heterochromatic *hmr* in the *rtt109*Δ and *msh2*Δ *rtt109*Δ strains. *ura3* mutations at heterochromatic *hmr* in the *rtt109*Δ and *msh2*Δ *rtt109*Δ strains are above and below the *URA3* open reading frame, respectively. Base substitutions are shown as capital red letters, 1-bp deletions are depicted as blue Greek delta letters, and 1-bp insertions are presented as green capital letters. A 2-bp deletion, a 3-bp deletion, and a 16-bp deletion are boxed, and complex mutations are underlined.
(PDF)
