# Supplementary material for: Involvement of DNA mismatch repair in the maintenance of heterochromatic DNA stability in Saccharomyces cerevisiae
Source: PLoS Genet. 2017 Oct 25;13(10):e1007074. doi: 10.1371/journal.pgen.1007074 (PMC5673234; doi:10.1371/journal.pgen.1007074)
Supplement: S1 Table — The data are shown as averages ± 1 S.D. (n≥ 4). (DOC) [file pgen.1007074.s001.doc]

| **Genotype** | **Resistance of the indicated *hmr::URA3* strains to 5-FOA (%)** |
| --- | --- |
| wild type | 102 ± 10 |
| *exo1∆* | 98 ± 10 |
| *msh2∆ exo1∆* | 117 ± 22 |
| *rev3∆* | 87 ± 7 |
| *msh2∆ rev3∆* | 90 ± 13 |
| *exo1∆ rev3∆* | 111 ± 7 |
| *mlh1∆* | 97 ± 14 |
| *pms1∆* | 100 ± 7 |
| *msh3∆ msh6∆* | 108 ± 6 |
| *msh3∆* | 99 ± 8 |
| *msh6∆* | 95 ± 5 |
| *pol2-4* | 112 ± 15 |
| *pol2-4 pms1∆* | 101 ± 15 |
| *rtt109∆* | 97 ± 15 |
| *msh2∆ rtt109∆* | 96 ± 9 |
| *hmr-E∆* | 1 ± 1 |
| *msh2∆ hmr-E∆* | 0.2 ± 0.6 |
