# Supplementary material for: Involvement of DNA mismatch repair in the maintenance of heterochromatic DNA stability in Saccharomyces cerevisiae
Source: PLoS Genet. 2017 Oct 25;13(10):e1007074. doi: 10.1371/journal.pgen.1007074 (PMC5673234; doi:10.1371/journal.pgen.1007074)
Supplement: S2 Table — The mutation spectra were obtained as described in Materials and Methods. (DOC) [file pgen.1007074.s002.doc]

| **Mutation type/class** | **Genotype** | | |
| --- | --- | --- | --- |
|  | wt | *msh2Δ* | *msh2Δ exo1Δ* |
| Short deletions | 7 | 26 | 24 |
| Short insertions | 2 | 6 | 6 |
| Complex mutations | 1 | 1 | 2 |
| G>T | 4 | 6 | 5 |
| C>G | 2 | 0 | 3 |
| C>T | 1 | 3 | 4 |
| T>C | 2 | 0 | 2 |
| G>A | 8 | 2 | 1 |
| A>C | 2 | 0 | 1 |
| T>G | 8 | 2 | 1 |
| G>C | 4 | 0 | 0 |
| A>T | 1 | 0 | 0 |
| T>A | 1 | 0 | 0 |
| A>G | 1 | 0 | 0 |
| C>A | 6 | 4 | 1 |
| Total | 50 | 50 | 50 |
