# Supplementary material for: Involvement of DNA mismatch repair in the maintenance of heterochromatic DNA stability in Saccharomyces cerevisiae
Source: PLoS Genet. 2017 Oct 25;13(10):e1007074. doi: 10.1371/journal.pgen.1007074 (PMC5673234; doi:10.1371/journal.pgen.1007074)
Supplement: S3 Table — The strains are BKDY155 (wild type) and its mutant derivatives. The difference between mutation rates marked a or b is not statistically significant (ap = 0.6 and bp = 0.15 in two-tailed Mann-Whitney test). 95% confidence intervals are in parentheses. (DOC) [file pgen.1007074.s003.doc]

| **Genotype** | **5-FOA^R^ mutation rate at**  **heterochromatic *hmr::URA3*** | |
| --- | --- | --- |
|  | Absolute rate  (x10^-8^) | Relative  rate |
| wild type | 60  (52 – 74) | 1 |
| *msh6*Δ* exo1*Δ* * | 470  (370 – 570) | 8 |
| *msh6*Δ* exo1*Δ* rev3*Δ | 250  (230 – 300) | 4 |
| *msh6*Δ *rev3*Δ* * | 160 ^a^  (100 – 330) | 3 |
| *msh6*Δ | 200 ^a^  (140 – 230) | 3 |
| *exo1*Δ | 260  (230 – 270) | 4 |
| *exo1*Δ *rev3*Δ* * | 81  (51 – 97) | 1 |
| *rev3*Δ | 40 ^b^  (20 – 60) | 1 |
| *msh3*Δ *exo1*Δ | 270  (130 – 320) | 4.5 |
| *msh3*Δ *exo1*Δ *rev3*Δ | 70  (55 – 110) | 1 |
| *msh3*Δ *rev3*Δ | 16 ^b^  (11 – 37) | 0.3 |
| *msh3*Δ | 66  (53 – 84) | 1 |
