# Supplementary material for: Involvement of DNA mismatch repair in the maintenance of heterochromatic DNA stability in Saccharomyces cerevisiae
Source: PLoS Genet. 2017 Oct 25;13(10):e1007074. doi: 10.1371/journal.pgen.1007074 (PMC5673234; doi:10.1371/journal.pgen.1007074)
Supplement: S4 Table — The mutation spectra were obtained as described in Materials and Methods. Each of the four spectra is composed of 50 mutations. 95% confidence intervals are in parentheses. (DOC) [file pgen.1007074.s004.doc]

| **Genotype** | **Rate (x 10^-8^)** | | | | | |
| --- | --- | --- | --- | --- | --- | --- |
|  | 1-bp insertions | Base substitutions | 1-bp deletions | Complex mutations | Other mutations | Total |
| wild type (with heterochromatic  *hmr::URA3*) | 1.2 | 48 | 8.4 | 1.2 | 1.2 | 60  (52-74) |
| *msh2Δ* (with heterochromatic  *hmr::URA3*) | 60 | 170 | 250 | 10 | 10 | 500  (430-570) |
| wild type (with euchromatic Chr V::*URA3*) | 0.4 | 5.5 | 1.1 | <0.2 | <0.2 | 7  (5-10) |
| *msh2Δ* (with euchromatic Chr V::*URA3*) | 21 | 135 | 99 | <5 | 5 | 260  (200-300) |
| *sir2Δ* (with euchromatic *hmr::URA3*) | 0.3 | 10.4 | 0.3 | <0.3 | 2.1 | 13  (9-17) |
| *sir2Δ msh2Δ* (with euchromatic *hmr::URA3*) | 24 | 108 | 162 | <6 | <6 | 300  (250-380) |
