# Supplementary material for: Involvement of DNA mismatch repair in the maintenance of heterochromatic DNA stability in Saccharomyces cerevisiae
Source: PLoS Genet. 2017 Oct 25;13(10):e1007074. doi: 10.1371/journal.pgen.1007074 (PMC5673234; doi:10.1371/journal.pgen.1007074)
Supplement: S1 Fig — ura3 mutations at heterochromatic hmr in the rtt109Δ and msh2Δ rtt109Δ strains are above and below the URA3 open reading frame, respectively. Base substitutions are shown as capital red letters, 1-bp deletions are depicted as blue Greek delta letters, and 1-bp insertions are presented as green capital letters. A 2-bp deletion, a 3-bp deletion, and a 16-bp deletion are boxed, and complex mutations are underlined. (PDF) [file pgen.1007074.s005.pdf]

```
1       ATGTCGAAAG CTACATATAA GGAACGTGCT GCTACTCATC CTAGTCCTGT TGCTGCCAAG    60

                                                       A
                                      T    A           G
61      CTATTTAATA TCATGCACGA AAAGCAAACA AACTTGTGTG CTTCATTGGA TGTTCGTACC   120
                            Δ                  C    A
                                               C

                                     Δ  A                            T
121     ACCAAGGAAT TACTGGAGTT AGTTGAAGCAT TAGGTCCCA AAATTTGTTT ACTAAAAACA   180
                                                          A       Δ    A
                                                                 ΔΔ
                                                                 ΔΔΔ
                                                                [ΔΔ]

181     CATGTGGATA TCTTGACTGA TTTTTCCATG GAGGGCACAG TTAAGCCGCT AAAGGCATTA   240
              T               Δ          T                     Δ
                             ΔΔΔ                               Δ

                    A
                    A  A                        C
241     TCCGCCAAGT ACAATTTTTT ACTCTTCGAA GACAGAAAAT TTGCTGACAT TGGTAATACA   300
        C              Δ     T
                      ΔΔ     T
                     ΔΔΔΔ

                                                        C
                                       G                C
                 T     G               A        A       T          C  T
301     GTCAAATTGC AGTACTCTGC GGGTGTATAC AGAATAGCAG AATGGGCAGA CATTACGAAT   360
                              Δ                        Δ

                                                 T
361     GCACACGGTG TGGTGGGCCC AGGTATTGTT AGCGGTTTGA AGCAGGCGGC AGAAGAAGTA   420
         Δ     A                                A

                                            T       A
                     T      A               T       G          T Δ
421     ACAAAGGAAC CTAGAGGCCT TTTGATGTTA GCAGAATTGT CATGCAAGGG CTCCCTATCT   480
                         A  C                                    Δ

481     ACTGGAGAAT ATACTAAGGG TACTGTTGAC ATTGCGAAGA GCGACAAAGA TTTTGTTATC   540

        A  Δ                                                    A
        CT Δ          A                                G        A
541     GGCTTTATTG CTCAAAGAGA CATGGGTGGA AGAGATGAAG GTTACGATTG GTTGATTATG   600
        C                            T

            G
601     ACACCCGGTG TGGGTTTAGA TGACAAGGGA GACGCATTGG GTCAACAGTA TAGAACCGTG   660
                                    Δ

               [ΔΔ]                T [ΔΔΔ]                           Δ CΔ
661     GATGATGTGGT CTCTACAGG ATCTGACATT ATTATTGTTG GAAGAGGACT ATTTGCAAAG   720
                                                  A   T

                                          GG
                                           A
721     GGAAGGGATGCTAAGGTAGA  GGGTGAACGT TACAGAAAAG CAGGCTGGGA AGCATATTTG   780
         [ΔΔΔΔΔΔΔΔΔΔΔΔΔΔΔΔ]                            A   A

781     AGAAGATGCG GCCAGCAAAA CTAA                                          840
```
